# Supplementary material for: Removal of organic contamination from wastewater using granular activated carbon modified—Polyethylene glycol: Characterization, kinetics and isotherm study
Source: PLoS One. 2024 Jul 10;19(7):e0304684. doi: 10.1371/journal.pone.0304684 (PMC11236163; doi:10.1371/journal.pone.0304684)
Supplement: S1 File — (DOCX) [file pone.0304684.s001.docx]

**Supporting Information (SI)**

**Removal of organic contamination from wastewater using granular activated carbon modified - Polyethylene glycol: Characterization, Kinetics and Isotherm study**

Leila Choopani^1^, Mohammad Mehdi Salehi^1^, Hossein Mashhadimoslem*^,2^, Mobin Safarzadeh Khosrowshahi^3^, Mashallah Rezakazemi*^,4^, Ali A. AlHammadi^5,6^, Ali Elkamel^2,6^, Ali Maleki*^,1^

*^1^Catalysts and Organic Synthesis Research Laboratory, Department of Chemistry, Iran University of Science and Technology, Tehran 16846-13114, Iran.*

*^2^ Chemical Engineering Department, University of Waterloo, Waterloo, Ontario N2L 3G1, Canada.*

*^3^Nanotechnology Department, School of Advanced Technologies, Iran University of Science and Technology, Narmak, Tehran, Iran.*

*^4^Faculty of Chemical and Materials Engineering, Shahrood University of Technology, Shahrood, Iran.*

*^5^Center for Catalysis and Separations, Khalifa University, P.O. Box 127788 Abu Dhabi, United Arab Emirates.*

^6^ *Department of Chemical Engineering, Khalifa University, Abu Dhabi, United Arab Emirates.*

**Corresponding authors*

[hmashhadimoslem@uwaterloo.ca](mailto:hmashhadimoslem@uwaterloo.ca) *(H. Mashhadimoslem)* [mashalah.rezakazemi@gmail.com](mailto:mashalah.rezakazemi@gmail.com) *(M. Rezakazemi),* [maleki@iust.ac.ir](mailto:maleki@iust.ac.ir) *(A. Maleki)*

**S1. A standard curve for diazinon**

**Figure S 1**. The standard Calibration curve for the determination of diazinon concentration in aqueous solution.

**S2. A standard curve for Amoxicillin**

**Figure S 2**. The standard Calibration curve for the determination of Amoxicillin Concentration

in aqueous solution.

**S3. A standard curve for Crystal Violet**

**Figure S 3**. The standard Calibration curve for the determination of Crystal Violet Concentration

in aqueous solution.
