# Supplementary material for: Removal of organic contamination from wastewater using granular activated carbon modified—Polyethylene glycol: Characterization, kinetics and isotherm study
Source: PLoS One. 2024 Jul 10;19(7):e0304684. doi: 10.1371/journal.pone.0304684 (PMC11236163; doi:10.1371/journal.pone.0304684)
Supplement: Attachment — Submitted filename: Comments.docx [file pone.0304684.s002.docx]

**Comments:**

Dear editor of “***PLOS ONE***”

Thank you for inviting me to review the manuscript titled “***Removal of organic contamination from wastewater using granular activated carbon modified - Polyethylene glycol: Characterization, Kinetics and Isotherm study***”. The manuscript was carefully studied. There are major comments, which are presented below. However, it's recommended to publish in your valuable journal.

1. The introduction is too long, it is better to reduce it to 800 words maximum.
2. Figure 1 needs reference
3. The resistance of the distilled (DI) water should be stated in the text*.*
4. In the results section, it is stated that "... employing GAC-PEG in the treatment of water may lead to enhanced ...", do not your test results conclusively indicate that GAC increases the removal efficiency over GAC-PEG?
5. Improve the title of Table 3. It is not appropriate to bring every specification of the table in the title
6. This manuscript has no literature review and a good literature review should be included.
7. Table 5 is not mentioned in the text, it should be described in the text.
